# Retrospective analysis of repair of finger soft tissue defect with free dorsal foot and toe flap

**DOI:** 10.1097/MD.0000000000045853

**Published:** 2025-11-21

**Authors:** Tao Cao, Lijuan Yao, Wenbo Meng, Xudong Hou, Linyun Shui

**Affiliations:** a Department of Microsurgery, The Linquan County People’s Hospital, Fuyang, Anhui Province, China.

**Keywords:** fibular free flap, finger soft tissue defect, wound repair

## Abstract

This study investigates the clinical effect of the great toe fibular free flap transplantation for the repair of the volar soft tissue defect of the finger. Retrospective observation was made on the efficacy of 6 patients who received toe and plantar flap repair for palmar soft tissue defects from January 2023 to June 2025. All the flaps survived. The color of the flaps was similar to the surrounding skin, the texture was soft, and the appearance was satisfactory. In the postoperative follow-up, the two-point discrimination of the transplanted flap was 5 to 9 mm, and the excellent and good rate was 100% according to the trial standard of the upper limb function evaluation of the Hand Surgery Society of the Chinese Medical Association. The donor site wound healed well, with no ulceration and no abnormal gait. The great toe fibular skin flap has the advantages of stable vascular anatomical position, simple operation, beautiful appearance and good feeling after operation, which is a good surgical method for repairing the skin and soft tissue defects of the palmar side of the finger and is worthy of clinical application.

## 1. Introduction

China is a country with developed handicraft industry. While the developed handicraft industry brings us precious wealth, the incidence of accidental finger injury is also increasing year by year. As the most flexible organ of human beings, fingers will increase the social burden while reducing the quality of life once they are damaged and deformed. For the repair of finger injuries, people have higher requirements for the recovery of appearance, joint range of motion and sensory function, in addition to the simple repair of wounds. Therefore, for the repair of this kind of injury, the choice of flap.^[1]^ It is not only required to maintain the activity of the original joint and the recovery of sensory function after transplantation, but also to consider that the color and texture of the transplanted flap should be close to the original normal tissue, so as to maximize the aesthetic recovery.^[2]^ Therefore, how to repair finger soft tissue defects has been the focus of microsurgical research.

Skin defect and phalangeal bone exposure are common in finger abdomen trauma. There are many commonly used flaps. The traditional distal pedicled flaps include cross-thoracic flap, cross-arm flap, abdominal pedicled flap and so on. Proximal pedicled flaps include digital proper artery retrograde island flap, adjacent finger flap, thenar flap, etc.^[3]^ After distal pedicle flap surgery, the limbs are in passive fixed position for a long time, and the pedicle needs to be cut off in the second stage. After the operation, the recipient area looks bloated and needs flap plastic surgery.^[4]^ After the operation, the blood supply and sensation of the recipient area are poor, and the tip of the affected finger is cold in the cold season, which is easy to cause frostbite or scald, so the flap is seldom used at present. The proximal pedicled flap has a wide range of anatomy, large trauma, and the donor site is in the hand, leaving scar contracture affecting finger function.^[5]^ With the development of microsurgical techniques, there are more and more reports on the transplantation of the fibular free flap of the great toe for the repair of finger pulp defects.

The metatarsal skin of the toe is very similar to the palmar skin of the finger, and the metatarsal skin of the toe perfectly replaces the palmar skin of the finger in terms of shape and texture, which makes the flap the best flap for repairing the skin and soft tissue defects of the finger pulp. Chaoqun Yuan et al^[6]^ treated 31 patients with finger soft tissue defect with free fibular toe flap, and the results showed that the shape and function recovery of the repaired finger were in line with the requirements of the patients, and the flap was relatively wear-resistant and the operation was relatively safe. Therefore, this study selected 6 patients with finger soft tissue defects treated in the Department of Microsurgery of our hospital since 2023 to observe and analyze the advantages and disadvantages of the free fibular flap of the toe and report them.

## 2. Materials and methods

### 2.1. Clinical data

Retrospective observation was made on the efficacy of 8 patients who received toe and plantar flap repair for palmar soft tissue defects from January 2023 to June 2025, 8 patients were enrolled, and according to the exclusion criteria, 6 patients were finally selected. Inclusion criteria: All patients suffered from finger pulp trauma and tissue defect; and All patients and their families agreed to participate in the study and signed the operation consent and informed consent. Exclusion criteria: Patients with soft tissue defects in other parts; Patients with diabetes mellitus and abnormal coagulation function before operation; Patients with mental disorder who could not cooperate with the operation; Patients with previous history of finger injury; and The complete data of the patients could not be obtained during the follow-up. This study was also approved by the institutional ethical review board of Linquan County People’s Hospital.

### 2.2. Surgical technique

#### 2.2.1. Wound treatment

After the patient was admitted to the hospital, the wound of the affected finger was thoroughly debrided in the emergency department to remove the contaminated and inactivated tissues, and the dominant digital artery, vein and digital nerve were freed and marked for use. The defect area of the wound was covered with vaseline gauze to properly protect the wound with skin and soft tissue defects. When there was no sign of infection in the wound 4 to 8 days after operation, the free flap of fibular side of the great toe was repaired. During the operation, the wound of the affected finger pulp was thoroughly debrided again, the necrotic tissue was removed, and the marked digital arteries, veins and nerves on the dominant side were found for standby.

#### 2.2.2. Flap design

According to the defect of the palmar side of the finger, the flap was designed on the pulp of the fibular side of the toe with the course of the fibular toe artery as the axis. The axis of the flap: along the body surface projection line of the fibular proper nerve and blood vessel bundle of the great toe, The perforating point of the cutaneous branch of the flap: along the axis, the perforating point of the cutaneous branch of the flap is located at the junction of the proximal end of the great toe; and Face: The distal end of the flap should not exceed the toe end, and the 2 sides should not exceed the center of the palm of the toe and the fibular surface.^[7,8]^

#### 2.2.3. Skin flap incision

The palmar skin and subcutaneous tissue were incised along the design line to expose the proper digital neurovascular bundle, and the flap and pedicle were incised sharply on the deep surface of the neurovascular bundle until the flap was punctured. Then cut the other side of the flap, pay attention to protect the subcutaneous superficial vein, free it to the proximal end as much as possible, loosen the tourniquet, stop bleeding thoroughly on the flap and the wound surface of the donor site, and observe the blood supply of the flap, then carefully ligate and cut off the neurovascular bundle of the proper toe, cut off the pedicle, and cut off the flap. To be anastomosed with the nerve and vascular bundle in the recipient area.^[9,10]^

#### 2.2.4. Repair of flap and donor site

The flap was sutured to the recipient site, the proper digital artery and the superficial subcutaneous vein were anastomosed to the proper digital artery and the dorsal digital vein of the injured finger with 10-0 noninvasive suture, and the proper digital nerve and the digital nerve were anastomosed with 9-0 noninvasive suture to reconstruct the skin sensation. The donor site wound: The foot incision could be extended and a full-thickness skin graft on the dorsum of the foot could be taken.

#### 2.2.5. Postoperative management

The patients were kept in bed for one week after the operation, with routine anti-inflammatory, anti-infection, analgesic and other symptomatic treatment, side light heat preservation, regular observation of changes in blood supply of the flap, elevation of the affected limb and keeping the affected finger dry and clean. The patient was strictly forbidden to smoke and given subcutaneous injection of low molecular weight heparin calcium. Two weeks after the operation, the suture was removed according to the healing condition, and the patients returned to the hospital regularly for reexamination after discharge.

### 2.3. Observation index

Half a year after the operation, the function of the affected finger was evaluated according to the trial standard of upper limb function evaluation of the Hand Surgery Society of the Chinese Medical Association,^[11]^ which was divided into 4 items: motor, sensory, appearance and working function. The full score of each item was 4 points, the total score was 16 points, the excellent score was 16 to 13 points, the good score was 12 to 9 points, the fair score was 8 to 5 points, and the poor score was <4 points. Half a year after the operation, the two-point discrimination and complications of the affected finger were examined at the same time. The normal two-point discrimination of the palmar side of the finger was 3 to 5 mm. If it was more than 1 cm, it proved that the prognosis of the nerve function of the affected finger after repair was poor.

## 3. Result

There were 6 patients in this group, including 5 males and 1 female, aged 11 to 53 years. The causes of injury were machine crush injury in 4 cases, heavy crush injury in 1 case, and machine twist injury in 1 case. Fingers: thumb 2, index finger 2, middle finger 1, ring finger 1. The size of the defect ranged from 1.5 cm × 1.0 cm to 3.5 cm × 2.5 cm, with exposure of the phalanx in some patients. The time from injury to admission was 30 minutes to 3 hours. Specific information is shown in Table 1.

**Table 1:** Summarized data.

Number of patient	Male	Female	
	5	1	
Median age (yr)	37 (11–53)		
Etiology	Machine strangle 1	Heavy rolling 1	Machine crush injury 4
Flap dimension (cm)	1.5 × 1.0–3.5 × 2.5		
Two point discrimination (mm)	5–9		
Mean fap thickness (mm)	6 ± 2 (4–8)		
BMI	24.4 ± 3.9 (18–32)		
Follow up (mo)	12 ± 6 (6–18)		

BMI = body mass index.

**Figure 1.**
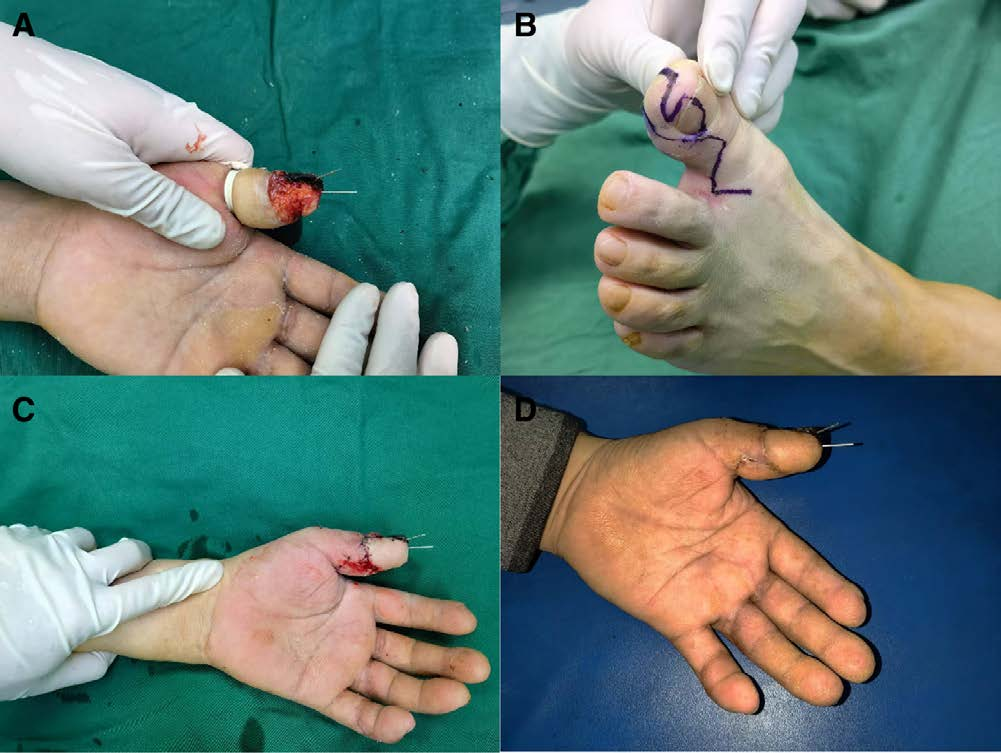
A patient with left thumb defect. (A) Preoperative condition; (B) flap design; (C) postoperative condition; (D) 1 mo after surgery.

**Figure 2.**
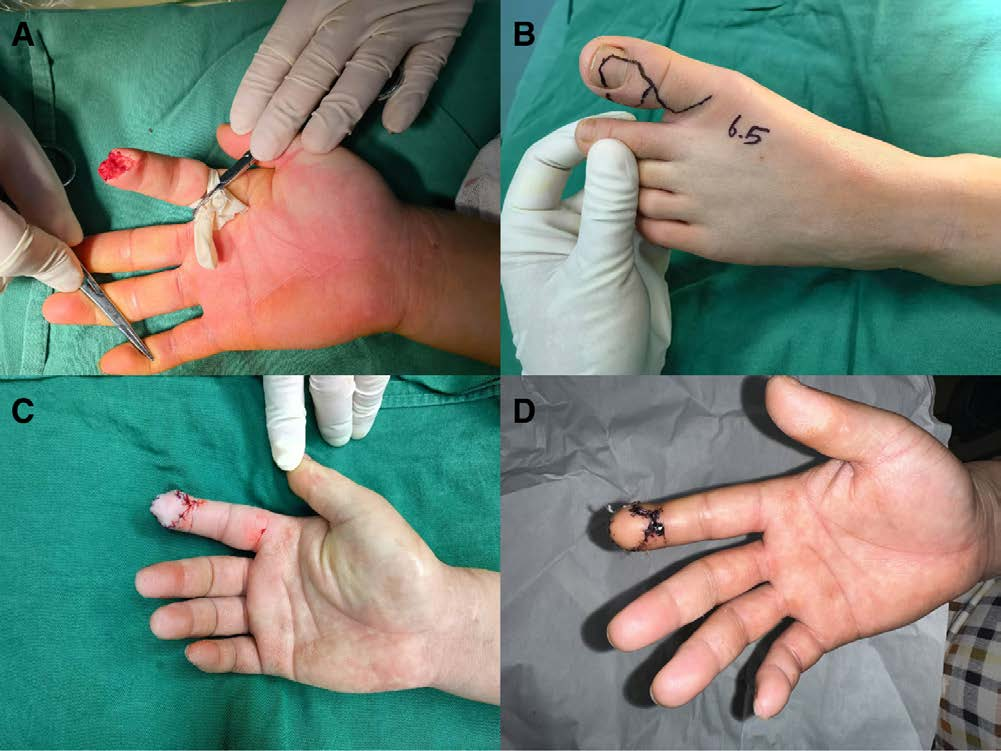
A patient with right index finger defect. (A) Preoperative condition; (B) flap design; (C) postoperative condition; (D) 1 mo after surgery.

All the flaps survived. The color of the flaps was similar to the surrounding skin, the texture was soft, and the appearance was satisfactory. The designed area of the flap was 1.5 cm × 1.0 cm to 3.5 cm × 2.5 cm, and the operation time was 2 to 3.5 hours. During the follow-up, the two-point discrimination of the transplanted flap was 5 to 9 mm, and the excellent and good rate was 100% according to the trial standard of the upper limb function evaluation of the Hand Surgery Society of the Chinese Medical Association. The total score of sensory function of the affected finger 6 months after surgical treatment is shown in Table 2. The donor site wound healed well, with no ulceration and no abnormal gait. Two typical patients were selected, as shown in Figures 1 and 2.

**Table 2:** Finger function recovery after 6 mo of surgical treatment.

Number of patient	Motor function	Sensory function	Working ability	Surface	Overall score
6	3.82 ± 0.31	3.80 ± 0.41	3.76 ± 0.61	3.65 ± 0.51	14.89 ± 0.95

## 4. Discussion

As a very important organ of the human body, the repair of finger defect not only needs to consider the recovery of function, but also the appearance after operation. Because the palmar soft tissue of the finger is thick, tough and sensitive, the selection of repair methods is highly required.^[12]^ At present, there are many surgical methods to repair the volar soft tissue defects of fingers in clinic, but the results after repair are different, and the long-term effects are quite different. Traditional distal flaps, such as cross-arm flap, abdominal pedicled flap, etc,^[3]^ need to be fixed for a long time after the operation, and the pedicle needs to be cut off in the second stage, and the flap is bulky after the operation. However, the traditional proximal flap, such as Aggag AM et al,^[13]^ has used the dorsal metacarpal artery flap to repair the skin and soft tissue defect on the palmar side of the finger. Although the operation is simple, the postoperative survival rate is high, and the donor area is considerable, the recovery of two-point discrimination after operation is not ideal. In recent years, there have been more and more reports on the use of the fibular flap of the great toe to repair the volar soft tissue defect of the finger. This flap is the best choice for repairing the volar soft tissue defect of the finger because of its similar structure with the volar skin of the finger, constant anatomical structure and moderate thickness.^[14]^

Through clinical application, the author believes that the flap has the following advantages: the flap is not bulky, the pulp of the finger is plump, and the shape is lifelike; the flap contains the digital nerve, the skin sensation is well restored, and the two-point resolution is high; the neurovascular bundle of the fibular flap of the toe is anatomically constant, the blood supply is reliable, and the flap is easy to cut; The diameter of the blood vessels in the donor site is almost the same as that in the recipient site, the anastomosis of the blood vessels is simple and the survival rate is high; The donor site of the flap was located on the fibular side of the toe, and the donor site of the skin graft could also be located on the same incision of the dorsum of the foot. Concealed, minimal damage; and postoperative flap with good elasticity, no pigmentation, wear-resistant. However, there are still some disadvantages: the flap is a free flap, which requires high skill of the surgeon; the flap may not be suitable for a large area of volar soft tissue defect of the finger; and It may take a long time for the donor site to recover after operation.

Although the operation is relatively simple, the following points should be paid attention to: The defect area of the wound should be carefully evaluated before the operation, and the shape of the flap should be reasonably designed. Traditionally, it is believed that the flap can provide a relatively limited area, but some scholars have reported that. The maximum size of the great toe fibular skin flap is 1.5 cm × 0.8 cm to 3.0 cm × 1.5 cm.^[15]^ The author believes that the size of the great toe fibular skin flap is relatively safe in the range of 1.0 cm × 0.5 cm to 1.5 cm × 1.0 cm, but the width of the flap should be <1.5 cm. The flap should be carefully removed, and the flap should not carry too much subcutaneous tissue to prevent the flap necrosis caused by the compression of the blood vessels in the flap after the operation; The vascular pedicle should be naked under the microscope, and the vascular injury can be reduced when the blood vessels are exposed clearly. Decrease in tissue volume can also avoid postoperative tissue swelling and compression of blood vessels; and When the flap is thinned microscopically, attention should be paid to protecting the proper toe nerve.^[16,17]^

However, this study also has some limitations, because our hospital is a county-level unit with a low level, and the limited number of cases may increase the chance of the results, also include additional limitations such as the single-site nature of the study, and lack of longitudinal data. We will continue to build departments to expand the influence in the future, and then such patients can seek medical treatment nearby, so as to reduce the waste of golden time for treatment and increase our experience.

## 5. Conclusion

To sum up, the fibular flap of the great toe has the advantages of constant vascular anatomical position, simple operation, beautiful appearance and good feeling after operation, which is a good surgical method for repairing the skin and soft tissue defects of the palmar side of the finger and compared with traditional repair methods, it is worth clinical promotion and application.

## Acknowledgments

We thank all colleagues in the Department of Microsurgery, the Linquan County People’s Hospital, for their help and support in this study.

## Author contributions

**Conceptualization:** Lijuan Yao.

**Data curation:** Lijuan Yao.

**Formal analysis:** Tao Cao.

**Investigation:** Linyun Shui.

**Project administration:** Wenbo Meng.

**Resources:** Wenbo Meng.

**Software:** Wenbo Meng.

**Supervision:** Linyun Shui, Xudong Hou

**Validation:** Linyun Shui, Xudong Hou.

**Visualization:** Xudong Hou.

**Writing – original draft:** Tao Cao.

**Writing – review & editing:** Tao Cao.
